# Preimplantation genetic testing in couples with balanced chromosome rearrangement: a four-year period real world retrospective cohort study

**DOI:** 10.1186/s12884-023-06237-6

**Published:** 2024-01-27

**Authors:** Fan Zhou, Jun Ren, Yutong Li, Yuezhi Keqie, Cuiting Peng, Han Chen, Xinlian Chen, Shanling Liu

**Affiliations:** 1 Department of Medical Genetics/Prenatal Diagnostic Center, West China Second University Hospital, Sichuan University, Chengdu, 610041 Sichuan China; 2 Key Laboratory of Birth Defects and Related Diseases of Women and Children (Sichuan University), Ministry of Education, Chengdu, 610041 Sichuan China

**Keywords:** Pre-implantation genetic testing, Balanced chromosome rearrangement, Blastocyst, Euploid

## Abstract

**Background:**

Couples with balanced chromosome rearrangement (BCR) are at high risk of recurrent miscarriages or birth defects due to chromosomally abnormal embryos. This study aimed to provide real-world evidence of the euploidy rate of blastocysts from couples with BCR using preimplantation genetic testing (PGT) and to guide pretesting genetic counselling.

**Methods:**

A continuous four-year PGT data from couples with BCR were retrospectively analyzed. Biopsied trophectoderm cells were amplified using whole genome amplification, and next-generation sequencing was performed to detect the chromosomal numerical and segmental aberrations. Clinical data and molecular genetic testing results were analyzed and compared among the subgroups.

**Results:**

A total of 1571 PGT cycles with 5942 blastocysts were performed chromosomal numerical and segmental aberrations detection during the four years. Of them, 1034 PGT cycles with 4129 blastocysts for BCR couples were included; 68.96% (713/1034) PGT cycles had transferable euploid embryos. The total euploidy rate of blastocysts in couples carrying the BCR was 35.29% (1457/4129). Couples with complex BCR had euploid blastocyst rates similar to those of couples with non-complex BCR (46.15% vs. 35.18%, *P* > 0.05). Chromosome inversion had the highest chance of obtaining a euploid blastocyst (57.27%), followed by Robertsonian translocation (RobT) (46.06%), and the lowest in reciprocal translocation (RecT) (30.11%) (*P* < 0.05). Couples with males carrying RobT had higher rates of euploid embryo both in each PGT cycles and total blastocysts than female RobT carriers did, despite the female age in male RobT is significant older than those with female RobT (*P* < 0.05). The proportions of non-carrier embryos were 52.78% (95/180) and 47.06% (40/85) in euploid blastocysts from couples with RecT and RobT, respectively (*P* > 0.05). RecT had the highest proportion of blastocysts with translocated chromosome-associated abnormalities (74.23%, 1527/2057), followed by RobT (54.60%, 273/500) and inversion (30.85%, 29/94) (*P* < 0.05).

**Conclusions:**

In couples carrying BCR, the total euploidy rate of blastocysts was 35.29%, with the highest in inversion, followed by RobT and RecT. Even in couples carrying complex BCR, the probability of having a transferable blastocyst was 46.15%. Among the euploid blastocysts, the non-carrier ratios in RecT and RobT were 52.78% and 47.06%, respectively. RecT had the highest proportion of blastocysts with translocated chromosome-associated abnormalities.

**Supplementary Information:**

The online version contains supplementary material available at 10.1186/s12884-023-06237-6.

## Background

Chromosome abnormalities, mostly balanced chromosome rearrangements (BCR), are found in approximately 1%–5% of couples with recurrent miscarriages [1]. Couples in which one partner carries a BCR may have an overall miscarriage rate as high as 49% due to unbalanced gametes [2]. Pre-implantation genetic testing (PGT) is performed before embryo transfer in vitro fertilization (IVF) and has been recommended to couples with BCR [3]. PGT is divided into two major categories for chromosome abnormalities detection based on the following indications: PGT for couples with chromosomal structural rearrangements (PGT-SR) and PGT for aneuploidy (PGT-A) detection in high-risk couples [3]. Blastocyst biopsy is currently the most widely used technique to obtain embryo samples [4]. A review study reported a live birth rate of approximately 28% per egg collection in 94,935 PGT cycles [5]. In another prospective study involving 8,137 human trophectoderm biopsies detected by next-generation sequencing (NGS), the overall euploid rate in PGT-A was found to be 43.3% [6].

Genetic counselling before PGT-SR is essential for couples with BCR. A significant concern for these couples is the possibility of obtaining transferable embryos. This information plays a crucial role in their decision-making process, as they may consider alternative methods such as artificial insemination by donor semen for male BCR carriers, oocyte donation for female BCR carriers, or even adoption. Furthermore, for couples with female diminished ovarian reserve (DOR) or those who do not have transferrable embryos after several PGT cycles, analyzing real-world datasets to determine the chance of obtaining a transferrable euploid embryo provides valuable reference information.

In theory, only couples with nonhomologous chromosome translocations or chromosome inversions have the potential to produce balanced or normal gametes, making them suitable candidates for PGT-SR. Among these, individuals with structural rearrangements involving three or more chromosomes or with three or more breakpoints are classified as having complex balanced chromosomal rearrangements (BCR) [7, 8]. Reciprocal chromosomal translocation (RecT), Robertsonian translocation (RobT), and inversion are classified into non-complex BCR. Genetic counseling for families with BCR considering PGT is challenging because the likelihood of having transferable embryos varies from the theoretical probability. Consequently, determining the specific proportion of transferable embryos in real-world datasets, particularly for different BCR subtypes and couples carrying complex BCR, provides valuable evidence for genetic counseling purposes.

## Methods

### Patients inclusion

We included all PGT-SR and PGT-A cycles performed in the Department of Medical Genetics/Prenatal Diagnostic Center, West China Second University Hospital, Sichuan University, from January 2019 to December 2022. We excluded PGT cycles for monogenic diseases because not all embryos underwent chromosomal structural or numerical aberration detection. We excluded couples who underwent PGT because of chromosomal deletions or duplications. This study involved human data collection and was performed in accordance with the Declaration of Helsinki. Informed consent was obtained from all participants.

### Data collection

We collected all basic information, high-resolution karyotypes (550 G-bands), assisted reproductive processes of the included couples, and genetic test results of all blastocysts. We divided all included PGT cycles into four groups according to the karyotype results: reciprocal translocation (RecT), Robertsonian translocation (RobT), inversion, and complex balanced chromosome rearrangement (complex BCR). Chromosomal polymorphisms included in ISCN 2020, such as inv(9)(p12q13), were considered normal karyotypes. All high-resolution karyotype results were obtained from a certified cytogenetic laboratory in our department. Only BCR carriers with at least three-fourths of the segments could be detected, which is larger than 4 Mb, to reliably identify unbalanced segregation products, which were indicative of PGT-SR. Embryo morphology was assessed as previously described. Cell numbers, fragmentation, blastomere size: ‘stage specific’ versus ‘non-stage specific,’ nucleation, cytoplasmic anomalies, spatial distribution of cells, and compaction are considered in embryo morphology rating [9, 10].

### Embryo biopsy

Embryo biopsy with intracytoplasmic sperm injection (ICSI) is preferred for PGT. After extensive culturing, some embryos developed into blastocysts. Blastocysts are composed of two different cell types: the outer trophectoderm (TE), which gives rise to extraembryonic tissues, and the inner cell mass, which gives rise to the fetus. According to the development rate, Embryos were biopsied at the blastocyst stage, which is days 5–7 post-ICSI, until the ICM was clearly visible. Biopsy was performed in a buffered medium (G-MOPS PLUS) with simultaneous ZP opening and TE cell excision on the day of full blastocyst expansion by a combination of mechanical detachment of several (5–10) TE cells [4]. The cumulus cells were carefully removed prior to ICSI to avoid potential maternal contamination of the biopsy samples. Time-lapse imaging systems with a closed culture system were used, and the morphological rating of embryos was evaluated according to the Gardner criteria [10]. The biopsied embryos were immediately transferred to the culture medium and cryopreserved individually.

### Genetic testing of biopsied samples

After biopsy, cells are washed and collected in small reaction tubes (containing lysis buffer) for whole genome amplification (WGA). All biopsied samples were amplified using the multiple annealing and looping-based amplification cycles (MALBAC) methods according to the manufacturer’s instructions in the same tube in which the sample was collected [11]. Initial quality analysis was performed using gel electrophoresis. The WGA products were subjected to barcoding (molecular indexing), adapter ligation, amplification, and library preparation. All libraries were sequenced on an NGS platform (MiSeq or NextSeq CN 500). Raw data produced after sequencing were processed by computational analyses and bioinformatics using algorithms provided by Yikon Medical to map and align the short sequence reads to a reference human genome sequence (GRCh37). Genome coverage, average read depth, and number of reads were analyzed. The recommended minimum number of valid reads was 1 M, the valid read GC content (%) was between 38 and 44%, and the CV (1000 K bin size) was below 0.12. Two independent professionals analyzed data, and discrepancies were adjudicated by a third professional. The resolution threshold of the in-house platform was 1 Mb for rearrangement-related chromosomes and 4 Mb for other chromosomes. We confirmed the ability of the in-house platform to detect chromosomal mosaicism by using the indicated mixing ratio of normal to abnormal cell lines. Mosaicism lower than 20% (< 1/5 cells) or higher than 80% (> 4/5 cells) was considered undifferentiable from technical noise; thus, < 20% of mosaics were classified as euploid and > 80% as aneuploidy [12].

### Carrier distinguishment detection

For couples who desired to choose a non-RecT or non-RobT carrier embryo, we used the Mapping Allele with Resolved Carrier Status (MaReCs) strategy to distinguish normal embryos from translocation carriers [13]. Translocation breakpoints in chromosome-imbalanced embryos were first identified at high resolution (~ 200 kb) by locating copy number changes. Informative SNP markers located within 1 Mb of the detected breakpoints were identified and used to identify the translocation-carrying allele using linkage analysis [14].

### Data analysis

SPSS software (version 21.0; IBM, Armonk, New York, USA) was used to analyze the data. Baseline information and genetic testing results of blastocysts were analyzed and compared among the different BCR subtypes. Measurement data are presented as median and interquartile range (IQR) for non-normal distributions. Enumeration data are presented as frequencies and ratios, and comparisons among groups were performed using the chi-square test. The significance level was set at bilateral α = 0.05.

## Results

### Basic information

**Fig. 1:**
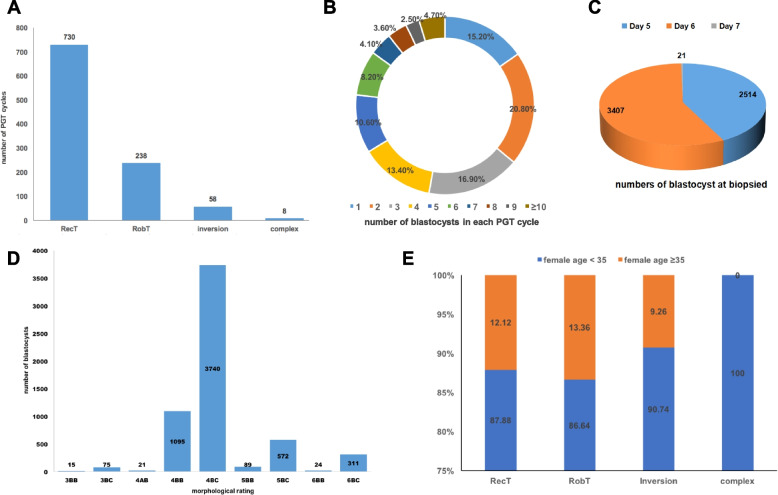
Baseline information of included PGT cycles. Panel **A**: The number of PGT cycles on RecT, RobT, inversion, and complex-BCR. Panel **B**: The number of blastocysts in each included PGT cycles. Panel **C**: The numbers of blastocyst biopsied on day 5, day 6, and day 7. Panel **D**: The numbers of blastocysts with different morphological ratings. Panel **E**: The proportion of female age < 35 years old and female age ≥ 35 years old in RecT, RobT, inversion, and complex-BCR. RecT, reciprocal translocation; RobT, Robertsonian translocation; BCR, balanced chromosome rearrangement

A total of 1571 PGT cycles with 5942 blastocysts were included for chromosomal numerical and segmental abnormality detection during the four years. Sixty-six percent (1034/1571) of the PGT cycles were performed in couples carrying BCR, and the remaining 34.18% (537/1571) were performed with PGT-A. As for each subgroup of couples carrying BCR, a majority of PGT cylces (730 cycles) were performed due to RecT, 238 PGT cycles were performed with RobT, and 58 cycles with inversion, seven couples with eight PGT cycles had chromosome rearrangements involving either three chromosomes or more than three breakpoints, which are referred to as complex BCR (Fig. 1A). The number of blastocysts per cycle ranged from 1 to 19, with a median number of 3 (interquartile range 2–5) (Fig. 1B). The total success rate of WGA using biopsied TE cells in this cohort was 99.23% (5896/5942). The numbers of embryos biopsied on days 5, 6, and 7 were 2514, 3407, and 21, respectively (Fig. 1C). Most embryos were rated as 4BC, followed by 4BB and 5BC (Fig. 1D). No statistical differences in the ratio of females aged > 35 years were found among the different subgroups of couples carrying non-complex BCR (*P*>0.05) (Fig. 1E).

Additionally, the female age demonstrated no significant differences among couples in RecT, RobT, inversion and complex BCR groups (*P*>0.05). However, the median years old of female age in PGT-A group is 35 (interquartile range 32–39), which is obviously older than the RecT, RobT, inversion and complex BCR groups (*P* < 0.05). For RobT subtype, female age in the male RobT subgroup is significantly older than those in the female RobT subgroup (*P* < 0.05) (Table 1). Meanwhile, couples in the male RobT group showed less times of spontaneous miscarriage compared with those in the female RobT group (*P* < 0.05). Meanwhile, the number of miscarriage before PGT in couples with RecT is significantly higher than those couples with RobT (*P* < 0.05). No statistical differences were observed on numbers of miscarriage before PGT in other comparisons (*P*>0.05) (Table 1).

**Table 1:** The pre-implantation genetic testing results of blastocysts from couples carrying non-complex BCR and complex BCR

	**Reciprocal translocation**			**Robertsonian translocation**			**Inversion**			**complex BCR**	**chi-q/Z**	***P-*value**
	**Male**	**Female**	**Total**	**Male**	**Female**	**Total**	**Male**	**Female**	**Total**			
**Female age (y)**^§^	30(28–33)	30(28–32)	30(28–32)	31(29–34)	30(28–33)	31(28.5–33)^*^	32(26.25–34)	30.5(28.75–34)	31 (27–34)	30 (28–32)	6.352	0.096
**No of miscarriage before PGT**^§^	1(0–2)	1(0–2)	1(0–2)	0(0–1)	1(0–2)	0(0–1)^*^	0(0–2)	1(0–2)	1(0–2)	1(0–2)	9.594	0.022^#^
**Euploid rate in each PGT cycle**^§^	0.25 (0.00–0.50)	0.25 (0.00–0.50)	0.25 (0.00–0.50)	0.50 (0.27–0.71)	0.40 (0.17–0.60)	0.50^*^ (0.21–0.67)	0.54 (0.23–0.96)	0.50 (0.13–1.00)	0.50 (0.19–1.00)	0.50 (0.30–0.59)	67.771	0.000^#&^
**Euploid rate in total**	30.47% (422/1385)	29.78% (464/1558)	30.11% (886/2943)	51.03% (247/484)	40.63% (180/443)	46.06% (427/927)^*^	61.32% (65/106)	53.51% (61/114)	57.27% (126/220)	46.15% (18/39)	130.326	0.000^#&∋^
**Abnormalities associated with TC**	71.24% (686/963)	76.87% (841/1094)	74.23% (1527/2057)	45.15% (107/237)	63.12% (166/263)	54.60%^*^ (273/500)	31.71% (13/41)	30.19% (16/53)	30.85% (29/94)	85.71% (18/21)	141.813	0.000^#&∋Δ^
**Abnormalities associated with both TC and other chromosomes**	29.88% (205/686)	31.51% (265/841)	30.78% (470/1527)	36.45% (39/107)	33.73% (56/166)	34.80% (95/273)	76.92% (10/13)	25% (4/16)	48.28% (14/29)	22.22% (4/18)	4.921	0.178
**Abnormalities unrelated to TC**	20% (277/1385)	16.24% (253/1558)	18.01% (530/2943)	26.86% (130/484)	21.90% (97/443)	24.49% (227/927)	26.42% (28/106)	32.46% (37/114)	29.55% (65/220)	7.69% (3/39)	35.206	0.000^&∋£^
**Mosaic rate**	4.26% (59/1385)	3.40% (53/1558)	3.81% (112/2943)	5.37% (26/484)	5.64% (25/443)	5.50% (51/927)	5.66% (6/106)	7.89% (9/114)	6.82% (15/220)	0 (0/39)	0.568	0.753
**Live birth in each PGT cycle**												
**One transplant**	51.56% (116/225)	57.83% (133/230)	55.96% (249/445)	55% (55/100)	61.90% (52/84)	58.15% (107/184)	45% (9/20)	38.10% (8/21)	41.46% (17/41)	57.14% (4/7)	3.858	0.271
**Two transplant**	45.83% (22/48)	42.42% (14/33)	44.44% (36/81)	45% (9/20)	39.13% (9/23)	41.86% (18/43)	40% (2/5)	60% (3/5)	50% (5/10)	100% (2/2)	2.399	0.506
**Three transplant**	0 (0/4)	50% (3/6)	30% (3/10)	50% (3/6)	100% (3/3)	66.67% (6/9)	100% (2/2)	0	100% (2/2)	0	4.063	0.120

*BCR* balanced chromosome rearrangement, *y* years old, *PGT* preimplantation genetic testing, *TC* translocated chromosomes

^§^Data was expressed with median (interquartile range, IQR)

^*^Significant differences are observed between male subgroup and female subgroup

^#^A significant difference was observed between the Robersonian and the reciprocal translocation groups

^&^Significant differences were observed between the inversion and reciprocal groups

^∋^Significant differences were observed between the inversion and Robersonian groups

^Δ^Significant differences were observed between the complex BCR and inversion groups

^£^Significant differences were observed between the complex BCR group and the other three groups

### Euploid rate of blastocysts

**Fig. 2:**
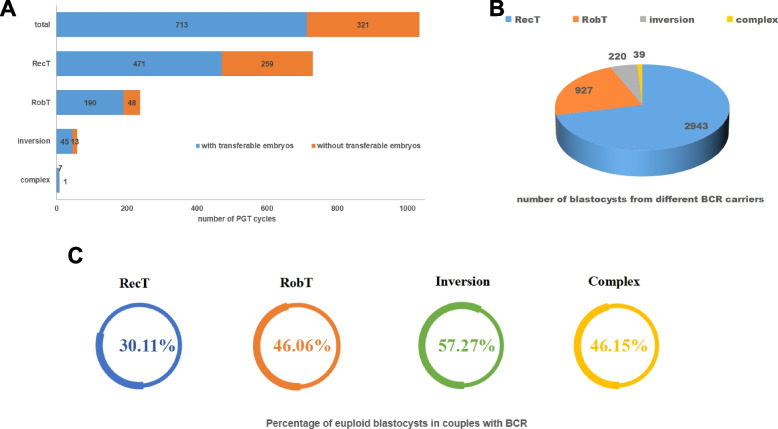
Number of PGT cycle, blastocysts, and percentage of euploid blastocysts. Panel **A**: The number of PGT cycles with or without transferable blastocysts. Panel **B**: The numbers of blastocysts in RecT, RobT, inversion, and complex-BCR. Panel **C**: The percentage of obtaining euploid embryos in biopsied blastocysts in RecT, RobT, inversion, and complex-BCR. RecT, reciprocal translocation; RobT, Robertsonian translocation; BCR, balanced chromosome rearrangement

Overall, 68.96% (713/1034) of the PGT cycles in PGT-SR had transferable euploid embryos, and 75.42% (405/537) of the PGT cycles in PGT-A had transferable euploid embryos. As for each subgroup, 471 PGT cycles of RecT (64.52%, 471/730), 190 PGT cycles of RobT (79.83%, 190/238), 45 PGT cycles of inversion (77.59%, 45/58) and seven PGT cycles of complex BCR (87.5%, 7/8) had transferable euploid embryos. Statistical differences were found among subgroups on the ratio of PGT cycles having tranferable embryos (*P* < 0.05) (Fig. 2A). Couples with RecT had significant lower chance to obtain transferable embryos per PGT cycles compared with RobT and inversion. Additionally, the euploid rate of blastocysts in each PGT cycle of couples with RecT is obviously lower than those couples with RobT (0.25 (0.00–0.50) versus (0.50 (0.21–0.67), *P* < 0.05). This euploid rate in the male RobT subgroup is also significantly higher than those in the female RobT group (0.50 (0.27–0.71) versus (0.40 (0.17–0.60), *P* < 0.05) (Table 1).

The numbers of blastocysts biopsied in couples with BCR are RecT (2943 blastocysts), RobT (927 blastocysts), inversion (220 blastocysts) and complex (39 blastocysts) (Fig. 2B). The total euploidy rate of blastocysts in couples carrying BCR was 35.29% (1457/4129) and 54.05% (980/1813) in couples with a normal karyotype. Couples with RobT had a higher chance of obtaining a euploid blastocyst than did those with RecT. In couples with RobT, 46.06% of the blastocysts were euploid, which was significantly higher than that in couples with RecT (30.11%) (*P* < 0.05). The ratio of euploid blastocysts in the inversion (57.27%) was significantly higher than that in the RecT (30.11%) or RobT (46.06%) (*P* < 0.05) (Fig. 2C). Moreover, significant differences were observed in the euploidy rate between female RobT (40.63%, 180/443) and male RobT (51.03%, 247/484) (*P* < 0.05). No statistically significant differences were found between male and female carriers of RecT or inversion (*P* > 0.05) (Table 1).

### Chromosomal abnormalities related to or unrelated to translocated chromosomes

As high as 74.23% (1527/2057) of chromosomal abnormalities in blastocysts from couples with RecT were associated with translocated chromosomes, which was significantly higher than those in RobT (54.60%, 273/500) (*P* < 0.05). Moreover, the translocation-associated aneuploidy rate in couples with inversion (30.85%) was significantly lower than that in RecT, RobT, and complex BCR couples (85.71%) (*P* < 0.05) (Table 1). Within the blastocysts with abnormalities associated with translocated chromosomes, 30.78% coexisted with chromosomal abnormalities unrelated to translocation in RecT couples, and the corresponding ratios in RobT and inversion couples were 34.80% and 48.28%, respectively (Table 1). Except for translocation-associated chromosomes, the mosaic rates of blastocysts in non-complex BCR ranged from 3.81% to 6.82% (Table 1).

### Couples with complex-BCR had a similar euploid rate of blastocyst compared with those couples with non-complex BCR

Eight PGT cycles of couples carrying complex BCR were analyzed. Cases 1, 6, and 7 involved two nonhomologous chromosomes with three or more breakpoints. Cases 2, 3, and 5 involved three nonhomologous chromosomes. Case 4 involved four nonhomologous chromosomes. GnRH antagonists and long agonists have been used to stimulate ovulation. A total of 39 blastocysts were biopsied and analyzed using NGS. Of these, 46.15% (18/39) of the embryos were euploid, demonstrating no significant difference compared to non-complex BCR (35.18%, 1439/4090) (*P*>0.05). Considering the subtypes of non-complex BCR, the euploid rate of blastocysts in complex BCR demonstrated no significant difference compared to those in RecT (30.11%, 886/2943), RobT (46.06%, 427/927), and inversion (57.27%, 126/220) (*P*>0.05).

Six couples had transferred blastocysts, and seven healthy babies were delivered (one with a twin pregnancy). A special case is a 27-year-old woman with karyotype results of 46, XX, der(6)(6 qter → 6q23.3::6p12 → 6q11::11p11.2 → 11 pter, der(11)(6 pter → 6p12::6q23.3 → 6q11::11p11.2 → 11 qter) in high-resolution (550 bands per haploid genome) G-band analysis combined with C-band staining. She also underwent chromosomal microarray analysis (CMA) to identify potential microdeletions or microduplications associated with chromosome rearrangements, and no copy number variations larger than 500 kb were detected. Six blastocysts were biopsied on day 5 (three 4BC embryos and one 4BB embryo) and day 6 (two 4BC embryos). Three of the six blastocysts were euploid, without chromosomal abnormalities. After embryo transfer, the patient had a monochorionic twin pregnancy. The patient underwent amniocentesis at 23 weeks of gestation, and amniofluid CMA results from both fetuses were normal. She had given birth to two healthy babies. More details on the blastocyst test results of the BCR couples are shown in Table 2 and supplementary Table 1.

**Table 2:** The karyotyping, clinical cycle, and prenatal diagnosis of couples carrying complex-BCR

	**karyotyping**	**ovulation stimulation**	**No of blastocyst/ eggs**	**Euploid embryos**	**Embryo transfer**	**Prenatal diagnosis**
Case 1	46, XX, der(6)(6qter → 6q23.3::6p12 → 6q11::11p11.2 → 11pter, der(11)(6pter → 6p12::6q23.3 → 6q11::11p11.2 → 11qter)	GnRH antagonist	6/24	3	yes	CMA (-) monochorionic twins, healthy
Case 2	46,XX,t(12;22;15)(p10;p10;q10)	GnRH antagonist	9/36	4	yes	CMA (-) singleton, healthy
Case 3	45, XX, der(13;14)(q10;q10)t(13;18)(q13;q22)	GnRH antagonist	3/4	0	N/A	/
Case 4	45, XX, der(13;14) (q10;q10);t(5;16)(q24;p13.1)	GnRH antagonist	2/10	1	yes	CMA (-); 46, X?,t(15;16)(q24;p13.1)mat, singleton, healthy
Case 5	46, XX, inv(2) (p12p22)t(15;22)(q15;q11.2)	GnRH antagonist	3/9	2	yes	CMA (-) singleton, healthy
Case 6	46,XX,t(1;6)(p31.1;q16.1);inv(6)(p21.3q16.1)	GnRH antagonist	4/9	1	yes	/
		long agonist	7/15	4	yes	CMA (-) singleton, healthy
Case 7	46,XY, inv(5)(q11.2q22)t(5;8)(q22;q11.2)	GnRH antagonist	5/15	3	yes	CMA + karyotyping (-) singleton, healthy

*BCR* balanced chromosome rearrangement, *CMA* chromosomal microarray analysis

### Embryo’s translocation carrier distinguishment

A total of 71 PGT cycles with 265 euploid blastocysts underwent MaReCs detection to distinguish the translocation carrier status of the transferable embryos. All PGT cycles resulted in two or more euploid embryos. Of these, 50 PGT cycles involved couples carrying RecT, and the remaining 21 PGT cycles involved couples carrying RobT. In total, 50.94% (135/265) of the embryos were non-carriers, and the remaining 47.55% (126/265) embryos were identified to carry chromosome translocations. No statistical difference was identified in the ratio of carriers to non-carriers in euploid embryos (*P* > 0.05). Fourty-six couples received a prenatal diagnosis, including cytogenetic analysis, and the results showed that MaReCs detection could reliably distinguish embryos carrying a balanced translocation from those with a normal karyotype (Table 3). Additional details of the blastocyst test results for MaReCs are shown in Supplementary Table 2.

**Table 3:** Couples received MaReCs detection and prenatal diagnosis of included PGT cycles

	**Reciprocal translocation**			**Robertsonian translocation**			**Chi-q**	***P-*value**
	**Male**	**Female**	**Total**	**Male**	**Female**	**Total**		
**Non-carrier rate of blastocyst**	54.17% (39/72)	51.85% (56/108)	52.78% (95/180)	40.68% (24/59)	61.54% (16/26)	47.06% (40/85)	0.756	0.385
**Prenatal diagnosis rate**	73.91% (17/23)	51.85% (14/27)	62% (31/50)	69.23% (9/13)	75% (6/8)	71.43% (15/21)	0.576	0.448

*MaReCs* Mapping Allele with Resolved Carrier Status, *PGT* preimplantation genetic testing

### Live birth rate of this cohort

A total of 713 PGT cycles produced euploid embryos. Follow-up data showed that 62.97% (449/713) had live births until Dec 1, 2023. The live birth rate in the first transplant cycle of single embryo transfer after PGT is 55.69% (377/677). The live birth rates in the second transplant cycle and third transplant cycle are 44.85% (61/136) and 52.38% (11/21), respectively (Table 1). No statistically differences were found among different translocation subgroups in the liver birth rate of the first, second and third transplant cycles (*P* > 0.05) (Table 1). Multiple elements determine the process of embryo implantation, and live birth lags behind embryo transfer by around 9 months. The live birth rate may not reflect the live birth rate after obtaining a fully euploid embryo using PGT-SR.

## Discussion

The ratio of transferable embryos in PGT-SR is important for decision-making in couples carrying BCR. Evidence suggests that a significant proportion of embryos formed by gametes from BCR carriers may lose or gain translocation-associated chromosomes [15, 16]. The specific proportions of balanced or normal gametes are influenced by factors such as the type of chromosome rearrangement, segregation pattern, carrier gender, and the number of chromosomes involved or underlying breakpoints [17–19]. Meiotic segregation modes of RecT, for example, include alternate segregation, adjacent-1 segregation, adjacent-2 segregation, 3:1 segregation, and 4:0 segregation [16]. Different sperm segregation patterns were observed in two brothers carrying the (7;8) (q11.21;cen) translocation [20]. Another study using fluorescent in situ hybridization (FISH) technology investigated the proportion of normal and abnormal sperm in 12 chromosome translocation carriers and reported that the most common meiotic segregation pattern of spermatozoa is alternate segregation, resulting in a ratio of 1:2 for normal sperm [21]. The incidence of unbalanced meiosis in sperm varies widely, ranging from 18 to 82% [22, 23]. While it is possible to estimate the proportion of balanced or normal gametes in males carrying chromosomal translocations based on semen analysis, it is nearly impossible to accurately predict the ratio of balanced egg production to normal egg production in female carriers, particularly for couples carrying complex BCRs. A real-world data analyses of euploid rate of biopsied blastocysts in PGT would provide essential information for the pretest genetic counselling.

This retrospective cohort study revealed that euploid blastocyst rates ranged from 30.11% to 57.27% in couples carrying BCR, the highest in those carrying inversion, and the lowest in those carrying RecT. The euploidy rates of blastocysts in different types of non-complex BCR in this study were slightly lower than those reported by Yuan P, which were 35.69% for RecT, 62.98% for RobT, and 71.95% for inversion [14]. Our study had a relatively large blastocyst sample size and used a continuous dataset with a lower risk of selection bias. Additionally, the euploidy rates of blastocysts from PGT-A in this study (54.05%) were slightly higher than those reported by Girardi L (43.3%) and Liu (41.61%) [6, 24].

Meanwhile, most published literature concentrates only on the ratios of whole-chromosome and segmental aneuploidy rates in PGT-SR without considering that chromosome abnormalities are related to translocated chromosomes. This study provides a detailed description of the ratios for generating translocated chromosome-associated abnormalities and abnormalities associated with both translocated and other chromosomes in different subtypes of BCR carriers. RecT had the highest chance of forming embryos with translocated chromosome-associated abnormalities, followed by RobT and inversion. Moreover, RobT had a higher incidence of blastocysts with chromosome abnormalities unrelated to balanced translocation than RecT, implying that RobT has a greater impact on ICE than RecT carriers do. Additionally, an observational study suggested an increasing incidence of chromosomal abnormalities unrelated to translocation, known as the interchromosome effect (ICE), in PGT embryos [25]. However, another study utilizing comprehensive aneuploidy screening techniques failed to establish this association [26].

This real-world data analysis also indicated that males carrying RobT had higher euploid rates both in each PGT cycles and total blastocysts than female RobT carriers did, despite the female age in couples with male RobT is significant older than those couples with female RobT. No corresponding differences were found between males and females carrying RecT. Our findings are in accordance with the evidence that meiotic segregation differs between male and female RobTs [27]. There is also evidence that the proportion of unbalanced sperm generated is typically lower than that of unbalanced oocytes because of more stringent cell cycle checkpoint mechanisms that reduce the production of unbalanced gametes in men [28].

In euploid embryos, the proportions of RecT and RobT non-carriers were 52.78% and 47.06%, respectively. These ratios were very close to the theoretical probabilities. Carriers of BCR mostly have a normal phenotype but can produce many different types of gametes during germ cell meiosis, and unbalanced gametes would lead to increased risks of infertility, recurrent spontaneous abortion, stillbirth, neonatal death or malformations, and even intellectual abnormalities in the offspring. MaReCs, a carrier distinguishment approach, can help a certain proportion of BCR couples select non-carrier embryos and reduce the transmission of balanced translocations from parents to offspring.

More importantly, couples with complex BCR, either three-way rearrangement carriers or double two-way translocations, had euploid blastocyst rates similar to those of RecT and RobT carriers. This result is markedly different from those reported in other studies [29]. Li et al. (2020) reported that as many as 90.91% (70/77) of the embryos were diagnosed as abnormal in couples with complex BCR [29]. Although blastomere and blastocyst biopsied samples were included, and the SNP array and NGS platform were used in the subsequent genetic testing, the dramatically different euploid ratios of embryos from couples with complex BCR remained unclear. The types of complex BCRs, mode of segregation, and even involved chromosomes would influence the ratio of euploid blastocysts. Our data conformed to the literature; during germ cell meiosis, the risk factors for type I complex-BCR with a simple 3-way or 4-way exchange of segments are similar to those for reciprocal translocations [30]. Accordingly, couples with a simple 3-way or 4-way exchange of segment translocations would benefit from PGT, as real-world data have shown that the probability of having a transferable embryo is similar to that of a non-complex BCR. However, it may be difficult to accurately calculate the theoretical ratio of euploid embryos during genetic counseling before PGT testing. With the development of high-resolution optical genome mapping, many complex BCRs have been identified. Larger real-world datasets are needed to provide more evidence on the ratio of euploid embryos in couples with complex BCR, thus providing evidence for genetic counseling.

This study has several advantages. We obtained complete comparative data of a real-world continuous PGT cycle cohort with a large sample size of different types of BCR. The MaReCs data, distinguishing the RecT or RobT carrier status of euploid blastocysts in 71 PGT cycles with 265 blastocysts, is the largest sample size reported to date. This study had some limitations. First, genetic testing of the blastocysts was performed using a standard NGS protocol. Although there is evidence that PGT based on NGS could improve clinical outcomes compared to SNP-based PGT [31], NGS-based PGT could not detect whole ploidy abnormalities or abnormalities below the predefined resolution. Second, we only concentrated on the genetic testing results of blastocysts and provided an overview of the euploid rate in blastocysts. We did not calculate the rate of embryos that developed into blastocysts per oocyte retrieved, considering that multiple factors influenced this process and was beyond the objective of this study.

## Conclusions

The euploid rates of blastocysts from couples carrying balanced chromosome rearrangements included complex BCR, ranging from 30.11% to 57.27%, the highest in inversion, followed by RobT and complex-BCR, and the lowest in RecT. In complex BCR carriers, the chance of obtaining a euploid-transferable blastocyst is similar with non-complex BCR, considering the total number of cases with complex BCR is small, the conclusions can only be drawn with caution. More data are needed to evaluate the worthwhile of applying PGT testing in these couples. Among euploid blastocysts, non-carrier ratios in RecT and RobT were 52.78% and 47.06%, respectively. RecT had the highest proportion of blastocysts associated with the translocated chromosomes with chromosomal abnormalities. Our large sample size data from the molecular genetics laboratory provide valuable information for genetic counselling before PGT testing for couples carrying BCR.

### Supplementary Information

**Additional file 1.**

**Additional file 2.**

## Data Availability

The datasets used and analysed during the current study are available from the corresponding author on reasonable request.

## Abbreviations

BCR: Balanced chromosome rearrangement
PGT: Preimplantation genetic testing
RobT: Robertsonian translocation
RecT: Reciprocal translocation
IVF: In vitro fertilization
PGT-SR: Preimplantation genetic testing for chromosomal structural rearrangements
PGT-A: Preimplantation genetic testing for aneuploidy
NGS: Next-generation sequencing
DOR: Diminished ovarian reserve
ICSI: Intracytoplasmic sperm injection
TE: Trophectoderm
WGA: Whole genome amplification
MALBAC: Multiple annealing and looping-based amplification cycles
MaReCs: Mapping Allele with Resolved Carrier Status
IQR: Interquartile range
CMA: Chromosomal microarray analysis
ICE: Interchromosome effect
